# Multisystem Inflammatory Syndrome in Adults or Hemophagocytic Lymphohistiocytosis: A Clinical Conundrum in Fully Vaccinated Adults With Breakthrough COVID-19 Infections

**DOI:** 10.7759/cureus.22123

**Published:** 2022-02-11

**Authors:** Hiba Narvel, Anahat Kaur, Jiyoung Seo, Abhishek Kumar

**Affiliations:** 1 Internal Medicine, Albert Einstein College of Medicine, Jacobi Medical Center, New York, USA; 2 Hematology and Medical Oncology, Albert Einstein College of Medicine, Jacobi Medical Center, New York, USA

**Keywords:** hemophagocytic lymphohistiocytosis [hlh], multisystem inflammatory syndrome in adults [mis-a], vaccination, hyperinflammatory syndrome, covid-19

## Abstract

Hyperinflammatory syndrome with breakthrough coronavirus disease 2019 (COVID-19) infection in a fully vaccinated patient is not a common finding. To the best of our knowledge, this is the first such case of a patient who received the Spikevax/Moderna (elasomeran mRNA-1273) vaccine. The patient exhibited clinical characteristics consistent with both multisystem inflammatory syndrome in adults (MIS-A) and hemophagocytic lymphohistiocytosis (HLH), thus posing a diagnostic challenge. Multi-inflammatory syndrome in COVID-19 patients is frequently seen in the pediatric population, but it is a rare entity in adults especially after receiving COVID-19 vaccination. The pathophysiology of MIS-A is not completely understood yet, but it is believed that this likely occurs due to antibody-mediated immune dysregulation. There is a possibility of enhanced serologic response in patients like ours who are vaccinated and have breakthrough COVID-19 infection, thus paving the way for overwhelming antibody-mediated immune activation. There is a significant overlap between symptoms of MIS-A and other hyperinflammatory syndromes such as HLH; hence, a high degree of clinical suspicion and thorough diagnostic workup is required to explore all differentials. Our case raises concerns regarding the lack of clear algorithms and guidelines to diagnose and manage MIS-A in adults post-COVID-19 vaccination.

## Introduction

Infection with severe acute respiratory syndrome coronavirus 2 (SARS-CoV-2) has been implicated as a trigger for many complex medical syndromes primarily driven by an enhanced inflammatory response. Several cases of hemophagocytic lymphohistiocytosis (HLH) occurring secondary to coronavirus disease 2019 (COVID-19) have been reported [1] and a new entity called multisystem inflammatory syndrome (MIS) has been described in the literature [2]. This entity was initially described in children (MIS-C) as a delayed occurrence after COVID-19 infection and was later also reported in adults (MIS-A) [2]. Several research studies have shown that vaccines for COVID-19 help prevent severe manifestations of the disease [3]. However, case reports are now surfacing where fully vaccinated individuals continue to develop significant complications [4]. Herein, we report one such rare case describing the initial presentation and management challenges in a fully vaccinated adult female with underlying untreated chronic lymphocytic leukemia (CLL)/small lymphocytic lymphoma (SLL) who developed breakthrough COVID-19 infection complicated by a hyperinflammatory syndrome.

## Case presentation

A 63-year-old female presented to the emergency department in August 2021 with a two-day history of bilateral leg weakness and left facial droop. She also reported feeling fatigued with subjective fevers, dry cough, diarrhea, and shortness of breath for a week. Her past medical history was significant for hypertension, type 2 diabetes, end-stage renal disease on dialysis, heart failure, and stroke. Past surgical history was notable for coronary artery bypass graft (CABG) and percutaneous coronary intervention (PCI). She lived with her daughter who was quarantined at home due to the SARS-CoV-2 infection. The daughter reportedly started feeling sick two weeks prior to the patient's admission at which time the patient likely got exposed. Although the patient was fully vaccinated against COVID-19, with two doses of Moderna vaccine (elasomeran mRNA-1273) four months ago, on admission, she was found to have positive SARS-CoV-2 polymerase chain reaction (PCR) from nasopharyngeal swab and reactive total SARS-CoV-2 antibody. The patient also had significantly elevated titers of COVID-19 spike antibody (>2,500 U/ml) showing an appropriate response to vaccination.

**Figure 1:**
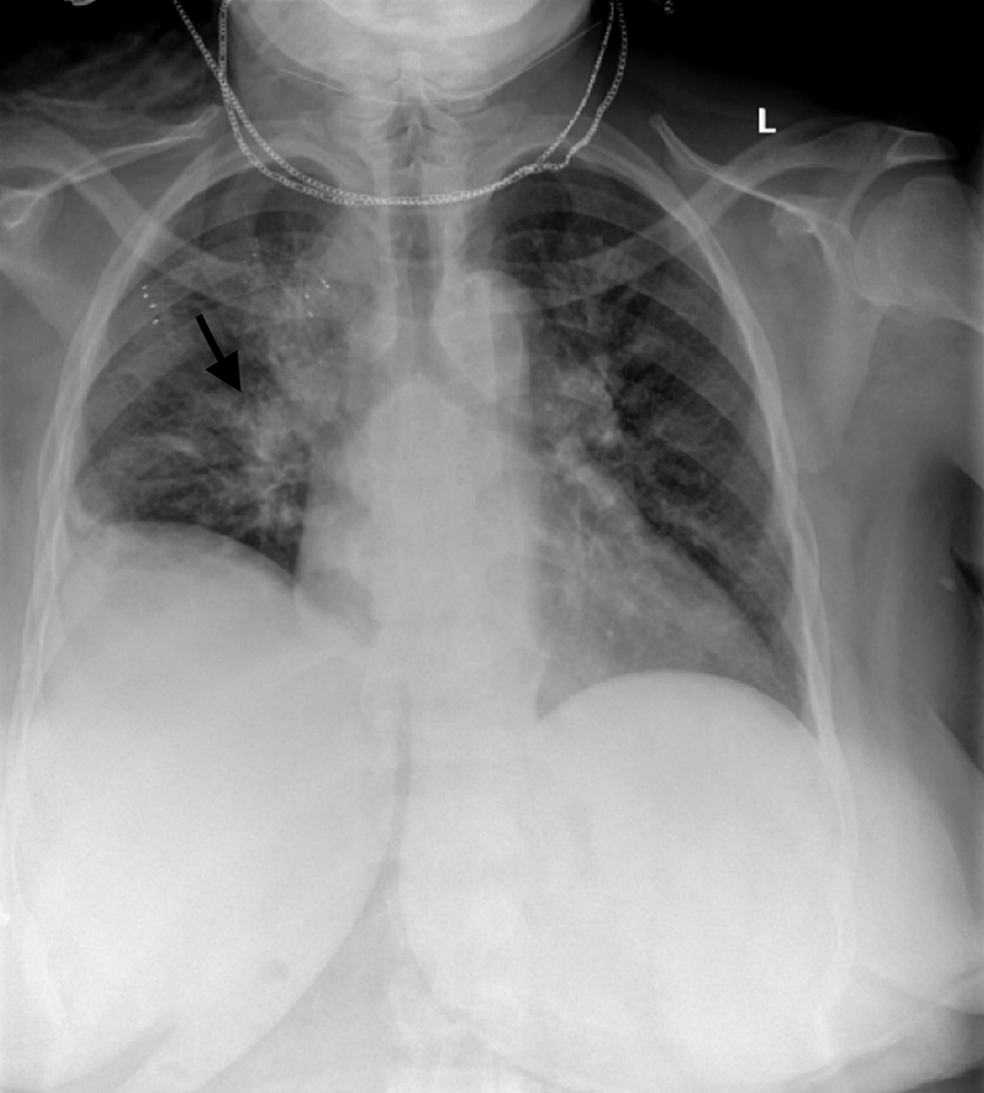
Chest X-ray (posteroanterior view) with right lung infiltrate (black arrow).

In the ER, she was afebrile, with blood pressure (BP) of 118/67 mmHg, respiratory rate (RR) of 21 breaths/min, heart rate (HR) of 73 beats/min, and normal oxygen saturation on room air. Decreased breath sounds over the right lung field were noted on physical exam and right-sided infiltrate was seen on chest X-ray (Figure 1).

Neurological examination was remarkable for mild flattening of the nasolabial fold on the left side, intact sensory examination in all four extremities, and mild bilateral leg weakness on motor examination (strength ⅘). Computed tomography (CT) scan of the head without contrast was done due to concern for neurologic deficits, which was unremarkable. Facial droop resolved spontaneously within 24 hours of admission and the patient was suspected to have a transient ischemic attack (TIA). On hospital day two, the patient was found to be in new-onset atrial fibrillation on ECG (sinus rhythm present on ECG done on day one), raising suspicion for a cardio-embolic event as a cause for TIA. The patient was started on apixaban and an echocardiogram was performed, which did not show any valvular vegetations or cardiac thrombi but did note decreased ejection fraction of 40% and left ventricular hypokinesis. Cardiac labs were noted to be abnormal with elevated troponin (2.270 μg/L) and pro-B-type natriuretic peptide (pro-BNP) (>70,000 pg/mL).

**Figure 2:**
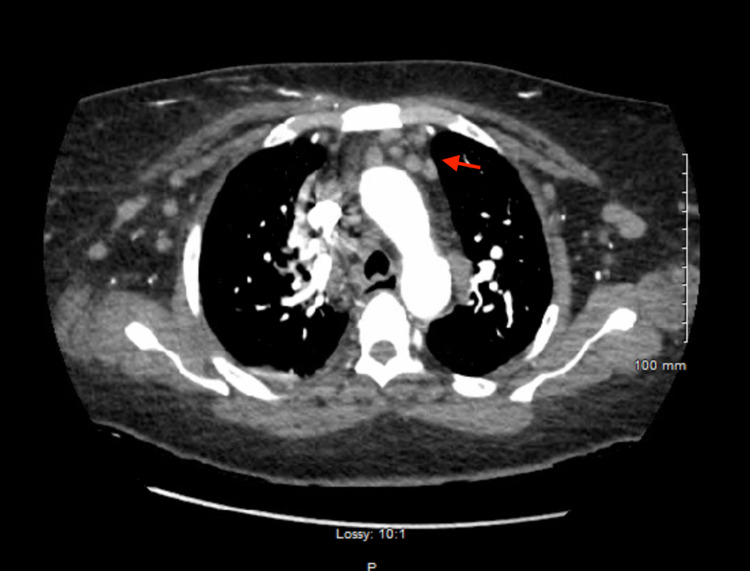
CT pulmonary angiography with contrast showing hilar and mediastinal lymphadenopathy (red arrow).

**Figure 3:**
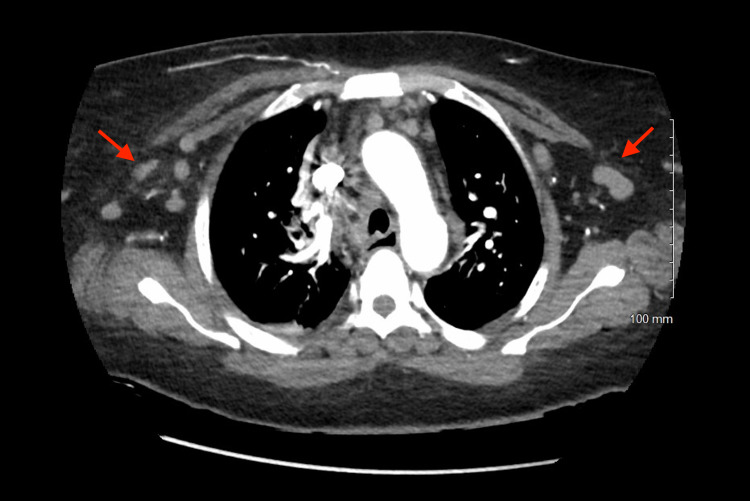
CT pulmonary angiography showing bilateral axillary lymphadenopathy (red arrows).

Initial complete blood count showed hypochromic, microcytic anemia (hemoglobin: 9.6 g/dL) and leucocytosis (41.66 white blood cells/nL) with 86.8% lymphocytes (36.15 lymphocytes/nL). Abdomen ultrasound depicted splenomegaly with spleen size 14.1 cm. CT pulmonary angiography with contrast showed no pulmonary embolism or focal consolidation but revealed multiple bulky bilateral axillary, hilar, and mediastinal lymph nodes raising suspicion for underlying hitherto undiagnosed lymphoproliferative disorder (Figures 2, 3). The right-sided infiltrate seen on the chest X-ray was not seen on the CT chest.

A peripheral blood smear was reviewed, which showed abundant mature-appearing small lymphocytes and smudge cells raising concern for CLL. Several left-shifted polymorphonuclear leukocytes with toxic granules were noted, which would be consistent with acute infectious processes. Flow cytometry showed aberrant B cells (79%), indeterminate for kappa and lambda, positive for CD19, CD23, CD5, and dim CD20, and negative for CD10, CD38, and FMC-7, which was suggestive of CD5+ lymphoproliferative disorder, likely CLL. Peripheral blood fluorescence in situ hybridization (FISH) CLL panel was normal.

The patient also had notably elevated inflammatory markers on admission with C-reactive protein (CRP) of 183.5 mg/dL and ferritin of 17,899 μg/L. Other abnormal labs included elevated aspartate transaminase (AST) of 681 U/L, alanine aminotransferase (ALT) of >700 U/L, D-dimer of 2,573 ng/mL, and procalcitonin (PCT) of 5.32 ng/mL on day one. Blood cultures, HIV, and hepatitis screening were negative.

The possible differential diagnosis for hyperinflammatory presentation included MIS-A, HLH, or macrophage activation syndrome (MAS). Severe COVID-19 infection was felt to be less likely since the patient did not have significant respiratory compromise and was maintaining normal oxygen saturation on room air. MAS was also less likely as the patient had no known diagnosis of an underlying rheumatologic condition. Testing along the lines of HLH was done, which showed elevated soluble interleukin-2 receptor level at 3,527 pg/ml (lab reference range 175-858 pg/ml) and elevated chemokine (C-X-C motif) ligand 9 (CXCL9) level at 6,000 pg/ml. Triglyceride (166 mg/dL) and fibrinogen (446 mg/dL) levels were within normal limits (Table 1).

**Table 1:** Remarkable initial lab values.

Component	Value
Hemoglobin	9.6 g/dL
White blood cells	41.66 cells/nL
Lymphocytes	36.15 cells/nL
C-reactive protein	183.5 mg/dL
Ferritin	17,899 μg/L
Aspartate transaminase	681 U/L
Alanine aminotransferase	>700 U/L
D-dimer	2,573 ng/mL
Procalcitonin	5.32 ng/mL
Soluble interleukin-2 receptor	3,527 pg/ml
Chemokine (C-X-C motif) ligand 9 (CXCL9)	6,000 pg/ml
Triglyceride	166 mg/dL
Fibrinogen	446 mg/dL

Bone marrow biopsy was deferred to avoid invasive procedure and limit the risk of exposure given the patient’s COVID-19-positive status.

The patient had no documented hypoxia and remdesivir for COVID-19 treatment could not be given in the setting of transaminitis. However, with abnormal chest X-ray (Figure 1) and elevated PCT, antibiotic therapy with ceftriaxone and azithromycin for five days was given for possible superimposed pneumonia. Due to concern for the hyperinflammatory syndrome, empiric treatment with steroids was started to suppress inflammatory response and prevent further organ dysfunctions. Treatment with intravenous immunoglobulin (IVIG), which has been utilized in patients with MIS-A, was not deemed to be necessary in this case since the patient was clinically improving.

**Figure 4:**
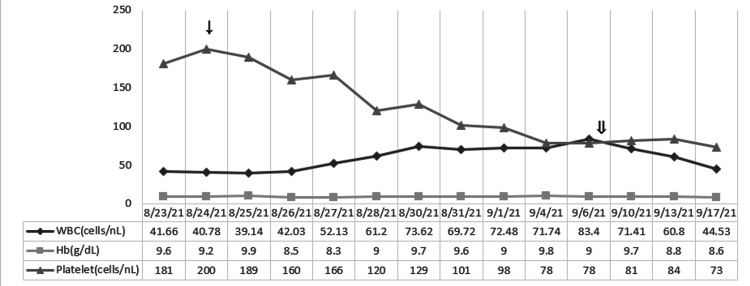
Response of the laboratory values of white blood cell (WBC), hemoglobin (Hb), and platelets with steroid taper treatment. ↓: starting dexamethasone 20 mg on 8/25; ⇓: starting dexamethasone 10 mg on 9/7.

**Figure 5:**
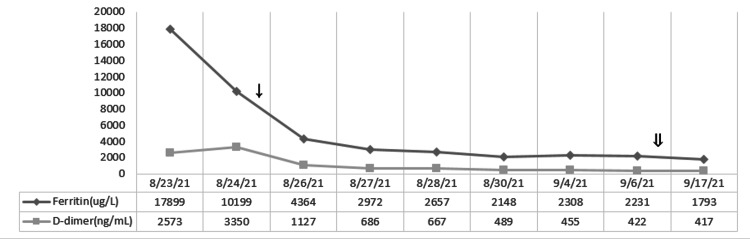
Response of the laboratory values of ferritin and D-dimer with steroid taper treatment. ↓: starting dexamethasone 20 mg on 8/25; ⇓: starting dexamethasone 10 mg on 9/7.

**Figure 6:**
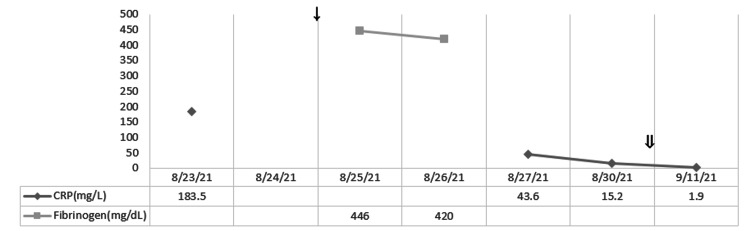
Response of the laboratory values of C-reactive protein (CRP) with steroid taper treatment. ↓: starting dexamethasone 20 mg on 8/25; ⇓: starting dexamethasone 10 mg on 9/7.

**Figure 7:**
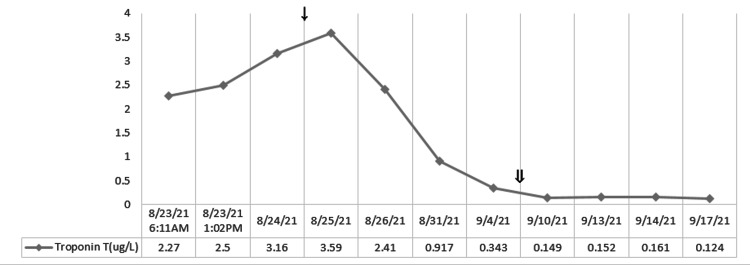
Response of the laboratory values of troponin T with steroid taper treatment. ↓: starting dexamethasone 20 mg on 8/25; ⇓: starting dexamethasone 10 mg on 9/7.

Rapid resolution of the patient's clinical symptoms and improvement in all laboratory values was noted over the next one to two weeks (Figures 4-7). The patient completed eight weeks of steroid taper, however, did continue to have prolonged viral shedding with positive COVID-19 PCR test at two-month follow-up when she was seen to discuss initiation of treatment for CLL.

## Discussion

Hyperinflammatory syndromes including MIS-A and HLH have been reported after infection with COVID-19 [5-9]. Cases are now coming to light where these severe complications continue to develop even in individuals who are fully vaccinated against COVID-19. Five cases of MIS-A and five cases of HLH post-vaccination have been reported until now, which are detailed in Tables 2, 3, respectively. Four cases of MIS-A were due to the Pfizer-BioNTech vaccine and one case was due to BBIBP-CorV (Sinopharm) vaccine [5-9].

**Table 2:** Reported cases of MIS-A occurring post-vaccination for COVID-19. MIS-A, multisystem inflammatory syndrome in adults; SARS-CoV-2, severe acute respiratory syndrome coronavirus 2; IL-6, interleukin 6.

Patient demographics	The duration between vaccination and onset of symptoms suggestive of MIS-A	Type of vaccine received	Symptoms	Labs	Authors
20-year-old Hispanic female	12 days after 1stdose of vaccine	Pfizer-BioNTech mRNA vaccine	Fever and rash for 3 days, with diarrhea, vomiting, cardiogenic shock, and acute renal failure	Elevated troponin and brain natriuretic peptide (BNP) with a left ventricular ejection fraction initially mildly reduced at 45% but 30-35% the following day	Salzman et al. [5]
40-year-old Hispanic man	42 days after 1st dose and 4 days after 2nd dose	Pfizer-BioNTech mRNA vaccine	Six days of episodic fevers up to 101.7°F. Associated symptoms included dyspnea on exertion, headache, neck pain, lethargy, abdominal pain, and diarrhea	Elevated inflammatory and cardiac markers	Salzman et al. [5]
18-year-old Asian American man	19 days after 1st dose	Pfizer-BioNTech mRNA vaccine	History of 3 days of fever as high as 104°F, with headache, vomiting, diarrhea, and abdominal cramping	Elevated inflammatory markers, thrombocytopenia, and lymphopenia. An echocardiogram revealed mild to moderate reduced systolic function with an ejection fraction of 40-45%	Salzman et al. [5]
44-year-old female; race unknown in the UK	2 days	Pfizer-BioNTech mRNA vaccine	Fever, upper arm pain, diarrhea, and abdominal pain over the next few days. She had an erythematous rash on the chest with subcutaneous edema	C-reactive protein (CRP) was 539 mg/L, white cell count of 17×109/L (1.8-7.5), troponin-T of 1,013 ng/L, and creatine kinase of 572 u/L	Nune et al. [6]
22-year-old male; unknown race in UAE	Started within a few hours, progressed over 4 days	BBIBP-CorV (Sinopharm) is an inactivated vaccine based on a SARS-CoV-2 isolate from a patient in China; it has an aluminum hydroxide adjuvant	High-grade fever, myalgia, nausea, vomiting, diarrhea, and a faint erythematous non-itchy rash over his torso that he noticed earlier that day	WBC count of 15,000, CRP level of 249 mg/ml, ferritin level of 4,357 ng/ml, D-dimer level of 14 mg/ml, procalcitonin level of 9 ng/ml, and IL-6 level of 90 pg/ml	Uwaydah et al. [7]

**Table 3:** Reported cases of HLH occurring post-vaccination for COVID-19. HLH, hemophagocytic lymphohistiocytosis.

Patient demographics	The duration between vaccination and onset of symptoms suggestive of HLH	Type of vaccine received	Symptoms	Labs	Authors
43-year-old Chinese female farmer	1 day after 1st dose	nCoV-19 vaccination (AstraZeneca)	Malaise, vomiting, persistent high fever	Pancytopenia, elevated triglyceride, decreased fibrinogen, increased transaminase and lactate dehydrogenase, high ferritin, low natural killer cell cytotoxicity	Tang and Hu [8]
60-year-old male	5 days after 1st dose	nCoV-19 vaccination (AstraZeneca)	Breathlessness, fevers, myalgia	Hemoglobin (10.1g/L), platelets (54/L), ferritin (159,076 μg/L), lactate dehydrogenase (536 iU/L), triglyceride (6.3 mmol/L), fibrinogen (0.7 g/L), alanine aminotransferase (132 iu/L), troponin (299 ng/L), and soluble CD25 (4,833 pg/mL). Bilateral pleural effusions on CT pulmonary angiography and severe left ventricular systolic dysfunction in 2nd echocardiogram	Attwell et al. [1]
Female in her 70s	7 days after 1st dose	nCoV-19 vaccination (AstraZeneca)	Night sweats, breathlessness, myalgia, persistent fever	Hemoglobin (11.9 g/L), platelets (69/L), ferritin (5,529 μg/L), lactate dehydrogenase (1,178 iU/L), triglyceride (2 mmol/L), fibrinogen (0.94 g/L), troponin (312 ng/L), and soluble CD25 (9,232 pg/mL). Right upper zone opacity in chest X-ray, bilateral patchy infiltrates in CT chest, and decreased left ventricular function in 2nd echocardiogram	Attwell et al. [1]
Male in his 30s	8 days after 1st dose	nCoV-19 vaccination (AstraZeneca)	Fever, diarrhea, sore throat, pruritic rash	Hemoglobin (10.5 g/L), platelets (319/L), ferritin (58,255 μg/L), lactate dehydrogenase (541 iU/L), triglyceride (2.7 mmol/L), fibrinogen (4.17 g/L), alanine aminotransferase (47 iu/L), troponin (42 ng/L), and soluble CD25 (3,575 pg/mL). Bilateral lung consolidation, pleural effusions, pericardial effusion, mild splenomegaly in CT chest, and decreased left ventricular systolic function in echocardiogram	Attwell et al. [1]
68-year-old man	18 days after 1st dose	nCoV-19 vaccination (AstraZeneca)	7 days of fevers, rigors, lethargy, night sweats	Sodium (125 mmol/L), elevated lactate dehydrogenase (854 U/L), low platelet count of 59 x 10^9^/L, elevated ferritin of 8,498 μg/L, normal fibrinogen of 2.3 g/L, elevated D-dimer of 10 mg/L, elevated aspartate aminotransferase of 223 U/L, and alanine aminotransferase of 121 U/L	Ai et al. [9]

All five reported cases of HLH were associated with the nCoV-19 vaccination (AstraZeneca) [1,8,9]. Clinical presentation and lab testing in our patient eventually met the criteria for both MIS-A and HLH, making it difficult to distinguish between these two conditions due to significant overlap. We report the first such case of hyperinflammatory syndrome in a patient with breakthrough COVID-19 infection after receiving the Moderna (elasomeran mRNA-1273) vaccine.

MIS-A is a febrile, hyperinflammatory syndrome, which generally manifests once seroconversion occurs two to six weeks after SARS-CoV-2 infection [10]. In most pediatric cases of MIS-C, patients initially experience an asymptomatic infection or mild COVID-19 symptoms, followed by the development of systemic inflammation a few weeks later, thus allowing for a contrast between the clinical presentation of acute COVID-19 and MIS-C. However, it has been found that 60% of adult patients with MIS-A had overlapping acute COVID-19 symptoms [10]. Our case falls into this category where the patient developed MIS-A-like presentation simultaneously after acute breakthrough COVID-19 infection. Like other cases of MIS-A post-vaccination, our patient had elevated inflammatory markers [5,6].

In addition to elevated inflammatory markers, our patient had several clinical features involving multiple organ systems that raised concern for MIS. She had prominent gastrointestinal symptoms like nausea and diarrhea on presentation, which are known to be highly prevalent in MIS-C and have also been reported in cases of MIS-A [11]. The patient’s presentation with elevated troponins, new-onset atrial fibrillation, and highly elevated N-terminal pro-B-type natriuretic peptide (NT-pro-BNP) suggested cardiac dysfunction, which is known to be a very common finding in MIS-C [12]. Neurologic abnormalities with transient lower extremity weakness and facial droop were also noted in addition to hepatobiliary dysfunction with abnormal transaminases. Severe COVID-19 infection alone could not explain the overall presentation in this case with multi-organ involvement but only mild respiratory symptoms.

Our patient met the case definition of MIS-A according to the CDC criteria (Table 4) and fit criteria for level 1 of diagnostic certainty (definitive case) based on Brighton Collaboration Case Definition [10]. Like the other cases of MIS-A post-vaccination that were reported by Salzman et al. and Nune et al., our patient responded well to steroids [5,6].

**Table 4:** Case definition of MIS-A. Multisystem inflammatory syndrome in adults (MIS-A) is diagnosed in a patient aged ≥21 years hospitalized for ≥24 hours, or with an illness resulting in death, who meets the listed clinical and laboratory criteria. The patient should not have a more likely alternative diagnosis for the illness (e.g., bacterial sepsis and exacerbation of a chronic medical condition) [10]. SARS-CoV-2, severe acute respiratory syndrome coronavirus 2; RT-PCR, reverse transcription-polymerase chain reaction.

Clinical criteria
Subjective fever or documented fever (≥38.0°C) for ≥24 hours prior to the hospitalization or within the first three days of hospitalization* and at least three of the following clinical criteria occurring prior to the hospitalization or within the first three days of hospitalization*. At least one must be a primary clinical criterion.
Primary clinical criteria
Severe cardiac illness Includes myocarditis, pericarditis, coronary artery dilatation/aneurysm, or new-onset right or left ventricular dysfunction (left ventricle ejection fraction < 50%), 2nd/3rd degree A-V block, or ventricular tachycardia. (Note: cardiac arrest alone does not meet this criterion).
Rash and non-purulent conjunctivitis.
Secondary clinical criteria
New-onset neurologic signs and symptoms include encephalopathy in a patient without prior cognitive impairment, seizures, meningeal signs, or peripheral neuropathy (including Guillain-Barré syndrome).
Shock or hypotension not attributable to medical therapy (e.g., sedation and renal replacement therapy).
Abdominal pain, vomiting, or diarrhea.
Thrombocytopenia (platelet count <150,000/ microliter).
Laboratory evidence
The presence of laboratory evidence of inflammation and SARS-CoV-2 infection.
Elevated levels of at least two of the following: C-reactive protein, ferritin, interleukin 6, erythrocyte sedimentation rate, and procalcitonin.
A positive SARS-CoV-2 test for current or recent infection by RT-PCR, serology, or antigen detection.
NOTE: *These criteria must be met by the end of hospital day three, where the date of hospital admission is hospital day 0.

The question was raised regarding the diagnosis of MIS-A being inconsistent with the patient’s clinical timeline of developing symptoms within 10 days of acute SARS-CoV-2 infection. Per the literature review, the timeframe of MIS-A manifestation after COVID-19 vaccination is variable. A report from NHS (UK) mentioned a case of MIS-A occurring within two days of vaccination [7]. We suspect that our patient’s MIS-A timeline could have been modified by the enhanced serologic response to breakthrough COVID-19 infection given prior vaccination history. Antibodies to SARS-CoV-2 usually develop two weeks after infection in most patients [13,14]. Our patient’s symptoms suggest the possibility of MIS-V (MIS due to vaccination), a condition previously described by Nune et al. where severe inflammation and subsequent multi-organ dysfunction develop within a week after an m-RNA, SARS-CoV-2 vaccine [6].

It is important to highlight that CLL patients have been noted to mount a sub-optimal antibody response to COVID-19 vaccination. In a study done by Herishanu et al., antibody response rates in treatment-naive CLL patients were significantly lower as compared to controls (55% vs. 100%) [15]. However, this does not appear to be the case with our patient who had elevated titers of COVID-19 antibodies on admission.

HLH precipitated by acute COVID-19 infection was considered an alternative working diagnosis in this case. Based on established diagnostic guidelines initially reported in the HLH 2004 trial, the patient met four out of nine criteria required for HLH diagnosis including splenomegaly, elevated ferritin, soluble interleukin-2 receptor level, and CXCL9 level (Table 5). One clinical feature that disputed a diagnosis of HLH was the absence of cytopenias on admission since these are generally seen in 80% of adult HLH patients on presentation [16]. Splenomegaly could also have been chronic in the setting of previously undiagnosed CLL/SLL and possibly not related to the acute presentation. Empiric treatment with steroids was started to suppress inflammatory responses and prevent further organ dysfunctions.

**Table 5:** Diagnostic criteria for HLH as established in the HLH-2004 trial. Adapted from [17]. HLH, hemophagocytic lymphohistiocytosis; IL-2, interleukin-2; CXCL9, chemokine (C-X-C motif) ligand 9.

The diagnosis of HLH may be established if the patient has:
A molecular diagnosis consistent with HLH: pathologic mutations of PRF1, UNC13D, Munc18-2, Rab27a, STX11, SH2D1A, or BIRC4
Or
Five of the following nine findings:
Fever ≥ 38.5°C
Splenomegaly
Cytopenias (affecting at least two of three lineages in the peripheral blood): hemoglobin < 9 g/dL (in infants <4 weeks: hemoglobin < 10 g/dL), platelets < 100 × 10^3^/mL, and neutrophils < 1 × 10^3^/mL
Hypertriglyceridemia (fasting, >265 mg/dL) and/or hypofibrinogenemia (<150 mg/dL)
Hemophagocytosis in bone marrow, spleen, lymph nodes, or liver
Low or absent natural killer cell activity
Ferritin > 500 ng/mL
Elevated soluble CD25 (α-chain of soluble IL-2 receptor)
Elevated CXCL9

MAS (also called secondary HLH) is characterized by the activation and proliferation of macrophages and T cells. The term MAS is used when a hemophagocytic syndrome develops in children with juvenile idiopathic arthritis (JIA) and other rheumatologic conditions such as Kawasaki disease. MAS is thought of as HLH in the setting of a rheumatologic disorder rather than as a separate syndrome [18]. It was felt to be less likely in this case since the patient did not have any known diagnosis of underlying rheumatologic or autoimmune condition.

Elevated concentrations of interferon-gamma (IFN-γ)-induced CXCL9 were found in our adult patient, which has been reported in MIS-C cases. Studies have suggested that IFN-γ, CXCL9, chemokine (C-X-C motif) ligand 10 (CXCL10), or any combination of these cytokines might be elevated in MIS. High CXCL9 levels in MIS-C patients have been associated with markedly elevated systemic inflammatory markers and multi-organ dysfunction, including cardiac and liver dysfunction, which was also seen in our patient [19]. Significant elevations in CXCL9 have also been demonstrated in patients with primary and secondary HLH due to the activation of the IFN-γ pathway, thus making it a non-specific marker in terms of distinguishing between MIS and HLH [18].

Since a sepsis/septic shock-like picture makes the diagnosis of hyperimmune syndrome less likely, it is important to clarify why we refute that the patient had pneumonia and sepsis/septic shock. The reasons are as follows: the right-sided infiltrate initially seen on the chest X-ray at the time of admission cleared on the CT pulmonary angiography, that was done on the same day as the chest X-ray (usually, clearance on imaging lags behind the clinical symptoms by weeks); CT pulmonary angiography showed mild pulmonary edema and no consolidation was seen; the patient demonstrated clear improvement in all clinical and labs parameters shortly after starting steroids, which would not have happened if she were in sepsis; she had organ failure without hypotension, pressor requirements, or elevated lactate, with low clinical suspicion of sepsis; and procalcitonin is reported to be elevated in HLH in multiple cases [20,21].

We stand with our diagnosis that the patient had a hyperimmune response due to COVID-19 infection in the setting of complete vaccination.

## Conclusions

Multisystem hyperinflammatory syndromes are now being reported as a concerning occurrence in patients with COVID-19 even after receiving full vaccination. MIS-A is a rare but important syndrome that can be difficult to distinguish from HLH, MAS, or severe COVID-19 infection due to significant overlap in clinical presentation and laboratory diagnostic findings; hence, requiring a low threshold of suspicion and detailed workup to evaluate all diagnostic possibilities. Our case highlights the need to urgently address several issues related to hyperinflammatory syndromes occurring in association with COVID-19. Firstly, preliminary MIS-A case definitions and testing algorithms need to be refined further as currently, these include many features, which are commonly seen in most inflammatory conditions leading to a paucity of clear diagnostic guidelines. Secondly, optimal treatment strategies need to be elucidated for the management of hyperinflammatory syndromes post-COVID-19 vaccination. IV immunoglobulin, steroids, and other immunomodulatory agents have shown clinical improvement in some suspected cases of MIS-A. Given the rarity of such syndromes in adults, conducting randomized trials may prove logistically challenging, and systematic reviews of individual reported cases may need to suffice to streamline recommendations for diagnosis and management. We hypothesize that human anti-interferon gamma monoclonal antibody, emapalumab, could potentially be utilized in adults with MIS/HLH-like presentations associated with COVID-19. Thirdly, we also need to investigate the correlation between pre-existing medical conditions such as cancer that could be potential predisposing factors for MIS-A or HLH in patients who contract COVID-19 infection or receive COVID-19 vaccination. Long-term follow-up and monitoring should be considered in affected patients. Finally, the immunological etiopathogenesis of MIS-A needs to be further clarified to determine whether vaccine-mediated serologic response could precipitate this hyperinflammatory syndrome with an earlier onset as compared to other MIS-A cases that occur after natural COVID-19 infection in unvaccinated individuals. This could have implications on the safety of COVID-19 vaccine administration and the need for vaccinating patients who already got COVID-19 infection.

## References

[1] Attwell L, Zaw T, McCormick J, Marks J, McCarthy H (2021). Haemophagocytic lymphohistiocytosis after ChAdOx1 nCoV-19 vaccination. [PREPRINT]. J Clin Pathol.

[2] Morris SB, Schwartz NG, Patel P (2020). Case series of multisystem inflammatory syndrome in adults associated with SARS-CoV-2 infection — United Kingdom and United States, March-August 2020. MMWR Morb Mortal Wkly Rep.

[3] Voysey M, Clemens SA, Madhi SA (2021). Safety and efficacy of the ChAdOx1 nCoV-19 vaccine (AZD1222) against SARS-CoV-2: an interim analysis of four randomised controlled trials in Brazil, South Africa, and the UK. Lancet.

[4] Rosenblum HG, Hadler SC, Moulia D (2021). Use of COVID-19 vaccines after reports of adverse events among adult recipients of Janssen (Johnson & Johnson) and mRNA COVID-19 vaccines (Pfizer-BioNTech and Moderna): update from the Advisory Committee on Immunization Practices — United States, July 2021. MMWR Morb Mortal Wkly Rep.

[5] Salzman MB, Huang CW, O'Brien CM, Castillo RD (2021). Multisystem inflammatory syndrome after SARS-CoV-2 infection and COVID-19 vaccination. Emerg Infect Dis.

[6] Nune A, Iyengar KP, Goddard C, Ahmed AE (2021). Multisystem inflammatory syndrome in an adult following the SARS-CoV-2 vaccine (MIS-V). BMJ Case Rep.

[7] Uwaydah AK, Hassan NM, Abu Ghoush MS, Shahin KM (2021). Adult multisystem inflammatory syndrome in a patient who recovered from COVID-19 postvaccination. BMJ Case Rep.

[8] Tang LV, Hu Y (2021). Hemophagocytic lymphohistiocytosis after COVID-19 vaccination. J Hematol Oncol.

[9] Ai S, Awford A, Roncolato F (2022). Hemophagocytic lymphohistiocytosis following ChAdOx1 nCov-19 vaccination. J Med Virol.

[10] Vogel TP, Top KA, Karatzios C (2021). Multisystem inflammatory syndrome in children and adults (MIS-C/A): case definition & guidelines for data collection, analysis, and presentation of immunization safety data. Vaccine.

[11] Feldstein LR, Rose EB, Horwitz SM (2020). Multisystem inflammatory syndrome in U.S. children and adolescents. N Engl J Med.

[12] Hékimian G, Kerneis M, Zeitouni M (2021). Coronavirus disease 2019 acute myocarditis and multisystem inflammatory syndrome in adult intensive and cardiac care units. Chest.

[13] Long QX, Liu BZ, Deng HJ (2020). Antibody responses to SARS-CoV-2 in patients with COVID-19. Nat Med.

[14] Kubota K, Kitagawa Y, Matsuoka M (2021). Clinical evaluation of the antibody response in patients with COVID-19 using automated high-throughput immunoassays. Diagn Microbiol Infect Dis.

[15] Herishanu Y, Avivi I, Aharon A (2021). Efficacy of the BNT162b2 mRNA COVID-19 vaccine in patients with chronic lymphocytic leukemia. Blood.

[16] George MR (2014). Hemophagocytic lymphohistiocytosis: review of etiologies and management. J Blood Med.

[17] Henter JI, Horne A, Aricó M (2007). HLH-2004: diagnostic and therapeutic guidelines for hemophagocytic lymphohistiocytosis. Pediatr Blood Cancer.

[18] Ravelli A, Davì S, Minoia F, Martini A, Cron RQ (2015). Macrophage activation syndrome. Hematol Oncol Clin North Am.

[19] Rodriguez-Smith JJ, Verweyen EL, Clay GM (2021). Inflammatory biomarkers in COVID-19-associated multisystem inflammatory syndrome in children, Kawasaki disease, and macrophage activation syndrome: a cohort study. Lancet Rheumatol.

[20] Bhattacharya D, Iyer R, Nallasamy K, Vaiphei K (2019). Haemophagocytic lymphohistiocytosis with pulmonary mucormycosis: fatal association. BMJ Case Rep.

[21] Abouleish Y, Ahmad Q, Marik P (2020). The hunt for hemophagocytic lymphohistiocytosis using procalcitonin. Chest J.

